# Hints on the Re-organization of the Navy, Including an Examination of the Claims of Its Civil Officers to an Equality of Rights

**Published:** 1845-03

**Authors:** 


*Hints on the re-organization of the Navy, including an Examination of the claims of its Civil Officers to an Equality of Rights.* New York; Wiley and Putnam, 1845.

This is a pamphlet of some seventy pages, without the name of its author upon the title page. It is a vigorous and manly protest against the injustice done to the civil officers in that department of the public service, written by one who is evidently well acquainted with the subject. The solemn truth is, there is too much deference paid to military as compared with civil services, not only by the government, but by the people of this country—an idolatry for which they *have* already suffered, and probably *will* suffer vastly more. Nothing so strongly demonstrates the weakness of the human mind as the stare of the multitude at a subaltern with a knot upon his shoulder, while the philosopher, philanthropist, or profound statesman, passes by unheeded. If the Officers of the Navy wish to have skilful surgeons to dress their wounds and prescribe for their diseases, they must unite in efforts to secure for them the pay and consideration which is due to gentlemen.

